# Implementation and optimization of SpMV algorithm based on SW26010P many-core processor and stored in BCSR format

**DOI:** 10.1038/s41598-024-67462-3

**Published:** 2024-07-17

**Authors:** Mengfei Ma, Xianqing Huang, Jiali Xu, Dongning Jia

**Affiliations:** 1 Information Research Department, Qingdao Marine Science and Technology Center, Qingdao, China; 2 Laoshan Laboratory, Qingdao, China; 3 College of Information Science and Engineering, Ocean University of China, Qingdao, China

**Keywords:** Sparse matrix–vector multiplication, Caching algorithm, SW26010P, Block compressed sparse row, High performance computing, Computer science, Software

## Abstract

The irregular distribution of non-zero elements of large-scale sparse matrix leads to low data access efficiency caused by the unique architecture of the Sunway many-core processor, which brings great challenges to the efficient implementation of sparse matrix–vector multiplication (SpMV) computing by SW26010P many-core processor. To address this problem, a study of SpMV optimization strategies is carried out based on the SW26010P many-core processor. Firstly, we design a memorized data storage transformation strategy to transform the matrix in CSR storage format into BCSR (Block Compressed Sparse Row) storage. Secondly, the dynamic task scheduling method is introduced to the algorithm to realize the load balance between slave cores. Thirdly, the LDM memory is refined and designed, and the slave core dual cache strategy is optimized to further improve the performance. Finally, we selected a large number of representative sparse matrices from the Matrix Market for testing. The results show that the scheme has obviously speedup the processing procedure of sparse matrices with various sizes and sizes, and the master–slave speedup ratio can reach up to 38 times. The optimization method used in this paper has implications for other complex applications of the SW26010P many-core processor.

## Introduction

**Figure 1:**
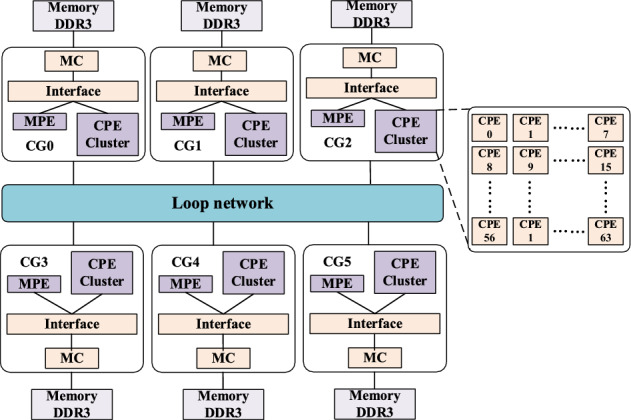
General architecture of the SW26010P many-core processor.

High-performance computing, also known as supercomputing, is a vital frontier branch of computer science. Since the birth of electronic computers, the improvement of computing performance has been one of the core goals pursued by information industry practitioners. It is not only a critical symbol of a country’s comprehensive scientific research level, but also provides the basis for national security, economic, social development and other sustainable development^1,2^. At present, the development trend of supercomputers is many-core and heterogeneous many-core. The computing capability of the new-generation Sunway supercomputer is provided by a SW26010P many-core processor that includes 6 core-groups (CGs), each of which includes one management processing element (MPE), and one 8 × 8 CPE cluster, these main components are connected through a ring network, as shown in Fig. 1. When the program is running, the main process runs on the MPE. MPE is essentially a general-purpose processor, in which complex computing departments can specify to CPE, each CPE is a lightweight core and performs a single thread^3^. MPEs mainly include computation, control, communication, and I/O functions. CPEs are mainly used for computation. A CPE and MPE are called a master core and slave core, respectively. The CPEs in the CGs are interconnected with one another and with external interactions through an intra-array network. Each CPE has an independent instruction cache and 256 KB local data memory (LDM) that is consisted of SPM. The LDM of CPE is identical to a cache that can improve data access speed^4,5^.

Sparse matrix–vector multiplication (SpMV) y = Ax is a fundamental computational kernel in scientific and engineering computing, which has been widely used in many fields such as fluid dynamics simulation calculation, data mining, graphics, and image processing^6,7^. The core of scientific and engineering computation lies in solving systems of linear equations. However, solving systems of linear equations is very time-consuming, and most of the computation in solving systems of equations is related to SpMV. Hence, the computational performance of SpMV often dramatically impacts the overall application time consumption^8,9^. In order to adapt the underlying architecture of hardware accelerators, researchers focus on the reconstruction of SpMV algorithm to improve the computing performance of hardware accelerators. Such as Intel Xeon Phi^10,11^, general purpose Graphics Processor (GPGPU)^12,13^, advanced micro devices (AMD)^14,15^, field programmable gate array (FPGA)^16,17^ and so on. With the advent of Sunway Taihulight supercomputer^18^, it is equipped with SW26010P many-core processor unique hardware architecture and has strong parallel computing capabilities. However, due to inconsistent hardware architecture, the optimization strategy of SpMV on GPU, AMD, Intel Xeon Phi and other processors cannot be applied to Sunway Supercomputer. The Sunway supercomputer is an important tool for solving major scientific problems and promoting technological innovation, mainly used for performing complex scientific calculations and simulations, such as climate models, physical experiment simulations, analysis of biomolecular structures, finite element analysis, computational fluid dynamics, circuit simulations, and so on. According to the literature^19–21^, SpMV is the core computational step in these applications; optimizing SpMV can significantly improve overall computational efficiency, thereby enhancing the Sunway supercomputer’s capability to solve major scientific problems. The computational efficiency of SpMV under the heterogeneous many-core architecture of Sunway Supercomputer is still one of the bottlenecks in scientific and engineering computing. It is very important to study the high-performance SpMV based on SW26010P many-core processor architecture. In addition, the underlying algorithm and software ecology of Sunway supercomputer are not perfect, and only by improving the computational efficiency of the underlying algorithm can we better meet the current computing performance needs. As the underlying algorithm of numerical simulation computing, SpMV optimization strategy based on SW26010P many-core processor plays a good role in promoting the improvement of domestic software ecology^22,23^. The sparsity properties of sparse matrices vary greatly. A large number of discrete indirect addressing operations are introduced in the calculation process of SpMV, which makes a large number of system resources consumed in accessing memory. How to realize SpMV to read matrix data efficiently and use the local memory of SW26010P many-core processor reasonably is the problem we focus on.

SpMV transplantation and optimization work has been uninterrupted, especially with the rapid upgrade of computing hardware, new research hotspots have followed. The optimization of sparse matrix storage format and the optimization in combination with the current computer architecture are the hot topics of recent research, such as optimizing SpMV based on many-core heterogeneous platforms. In terms of optimization of sparse matrix storage format, Kreutze et al. proposed a new storage format SELL-C-σ mainly from the perspective of improving vectorization performance, which effectively improves the storage efficiency and computational performance^24^. Liu et al. proposed a storage format CSR5 to improve the computation performance of irregular sparse matrix SpMV and conducted experiments on several heterogeneous many-core processors^25^. At the same time, he compared with the existing similar works, which showed an excellent optimization effect. Liu et al. analyzed the impact of different storage formats on SpMV and proposed an automatic SpMV tuning device SMAT, which can select and return the optimal storage format according to the characteristics of the specified sparse matrix to achieve the performance improvement of SpMV^26^. Bian et al. proposed a new sparse matrix storage format CSR2, which is a new single format for processor platforms with SIMD (single instruction multiple data) vectorization that has low complexity in transformation computation^27^. In terms of many-core heterogeneous platform optimization, Liu et al. proposed a parallel algorithm of SpMV based on CSR storage format based on SW26010 many-core processor, which was designed from the aspects of task division, LDM space division and adaptive excellence^28^. They innovatively proposed a dynamic and static buffer caching mechanism and a combination of dynamic and static task scheduling methods to improve the performance further. Li Y et al. took SW26010 as the platform to design and optimize the fine-grained parallel algorithm for SpMV at the thread level and instruction level parallel level, and conducted experiments on a large number of test sets, obtaining an average master–slave speedup ratio of 11.7 times^29^. Xiao et al. proposed a CASpMV based on the Sunway many-core processor for three main performance-limiting reasons storage limitations, load imbalance and irregular memory accesses^30^. They demonstrated the effectiveness and optimization efficiency of this work through extensive experiments. Sun et al. proposed an efficient SpMV calculation method SWCSR-SpMV for SW26010 processor^31^. They designed a dynamic preprocessing scheme to avoid the DMA reading operation to load useless x, reduce redundant memory access, and divide the many-core into smaller communication ranges. To share the public data on the worker thread through high-speed data bus, and through a lot of experiments to prove that the scheme has a good optimization performance. Yca B et al. proposed a two-segment large-scale SpMV, called tpSpMV, and introduced a two-stage parallel execution technology for tpSpMV to solve the limit of computing scale, and proposed an adaptive partitioning method and parallelization design to alleviate the problem of high memory access delay^32^. The experimental results on Sunway Taihulight supercomputer show that the proposed scheme has an obvious acceleration effect.

To improve the underlying algorithm ecology of Sunway supercomputer, enhance the computational performance and speed of numerical simulation, a SpMV algorithm based on BCSR storage is designed in this paper. The rest of the paper is organized as follows. In “Proposed method” section describes the design and implementation of the program. In “Experiments” section presents the experimental setting, results and discussion. Finally, the paper concludes in “Conclusions” section.

## Proposed method

### Overall design

Considering the unique structure of SW26010P many-core processor and the special storage format of BCSR, we designed SpMV algorithm based on BCSR storage format for SW26010P many-core processor. The BCSR storage format is a method for storing sparse matrices, with advantages such as memory efficiency, computational efficiency, parallelism, reduced memory access conflicts, and ease of implementation. These characteristics align with the architectural features and optimization goals of Sunway processors, making the choice of BCSR storage format on Sunway processors aimed at maximizing performance and efficiency. In terms of memory efficiency, it is an improvement over CSR; BCSR stores the matrix by dividing it into blocks of a fixed size, which reduces gaps in memory and increases memory utilization, significantly reducing the number of times vector x is fetched during computation. Regarding computational efficiency and parallelism, the BCSR format is suitable for parallel computing because it allows multiple threads or cores to process different matrix blocks simultaneously, thereby improving parallelism. In terms of reducing memory access conflicts, due to the contiguous storage of blocks in BCSR, it can reduce conflicts between cache lines, improving cache efficiency, and the effect of BCSR is more pronounced in large-scale matrices with local density.

**Figure 2:**
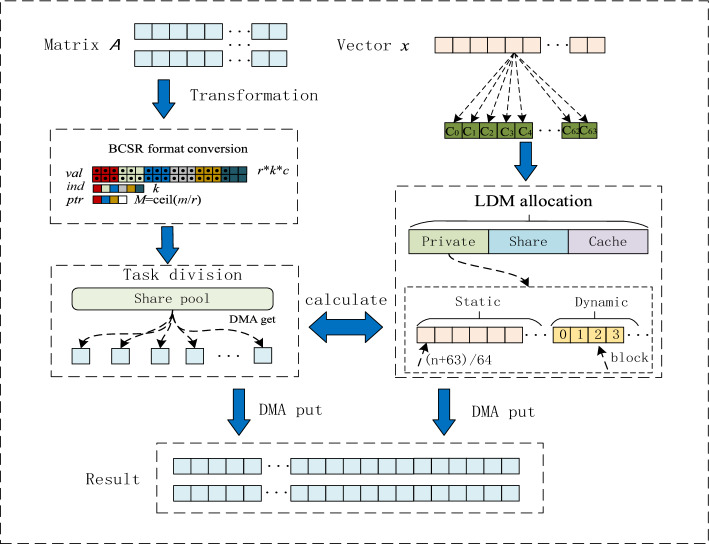
The SpMV algorithm implementation model.

The algorithm model mainly comprises the following modules: storage format conversion module, task division module, LDM allocation module, calculation module, data verification module, and so on. The primary function of the storage format conversion module is to quickly convert the large-scale sparse matrix of CSR storage format into BCSR format according to the computing requirements, and make a preliminary preparation for efficient SpMV computation. The primary purpose of the task division module is to distribute computing tasks to 64 CPEs as much as possible to achieve load balancing and improve computing efficiency. Therefore, this paper adopts the dynamic partition strategy to achieve load balancing. The specific implementation method is to divide the computation tasks into the same block and put it into the task pool established in advance. Each CPE obtains the computation data from the task pool in turn. At the same time, the problem of resource conflict between processes is avoided by locking. The main purpose of the memory allocation module is to use the memory of SW26010P many-core processor properly. Due to the LDM space memory limit of SW26010P many-core processor, we have refined the LDM memory allocation of CPE to achieve the calculation performance of (SpMV) y = Ax. The computing module and data verification module are the calculation of tasks and verification of data correctness. The algorithm implementation model is shown in Fig. 2.

### Converting storage formats

#### Implementation of format conversion

This paper is based on the storage format of CSR to achieve BCSR format conversion to achieve SpMV optimization. Therefore, the first step in this paper is to convert the large-scale sparse matrix in CSR storage format into BCSR format for storage. In the BCSR format, *val* stores nonzero elements stored in a block in a row-first fashion, *ind* stores the index of the first nonzero element in the block, and *ptr* stores the starting index of the first block in *ind* for the current row-slice. Firstly, the non-zero elements of the input large-scale sparse matrix are scanned, and the distribution of non-zero elements is analyzed to obtain the best size of BCSR data block block_size*block_size. The number of existing data blocks in the sparse matrix *block_n* is counted by the row-slice *ls*_*i*_. Record the starting column label *block_col*_*i*_ for each block. We call the number of the block in the slice of the current row *ls_block*_*i*_, where *i* ∈ [0, *ptr*[*i* + *1*]-*ptr*[*i*]]; Further, we iterate over the data in the original CSR format to obtain the row number *data*_*row*_ and column number *data*_*col*_. Calculate the block number *block_i*, the row number *block_in*_*row*_, and the column number *block_in*_*col*_ of the data block where the current nonzero element should be stored by scanning the matrix. Where *block_i*, *block_in*_*row*_ and *block_in*_*col*_ are calculated according to (1), (2) and (3):

1 $$block\_i = ptr\left[ {data_{row} /block\_size} \right] + ls_{\_} block_{i}$$

2 $$block\_in_{row} = data_{row} { - }block\_size*ls_{i}$$

3 $$block\_in_{col} = data_{col} block\_col_{[block\_i]}$$

The above values of *block_i*, *block_in*_*row*_ and *block_in*_*col*_ can be used to obtain the *val*_*index*_ of *val* corresponding to BCSR and assign the value. The calculation formula is shown in (4).

4 $$val_{index} = \left[ {\left( {block\_i*block\_size} \right) + block\_in_{row} } \right]*block\_size) + block\_in_{col}$$

**Figure 3:**
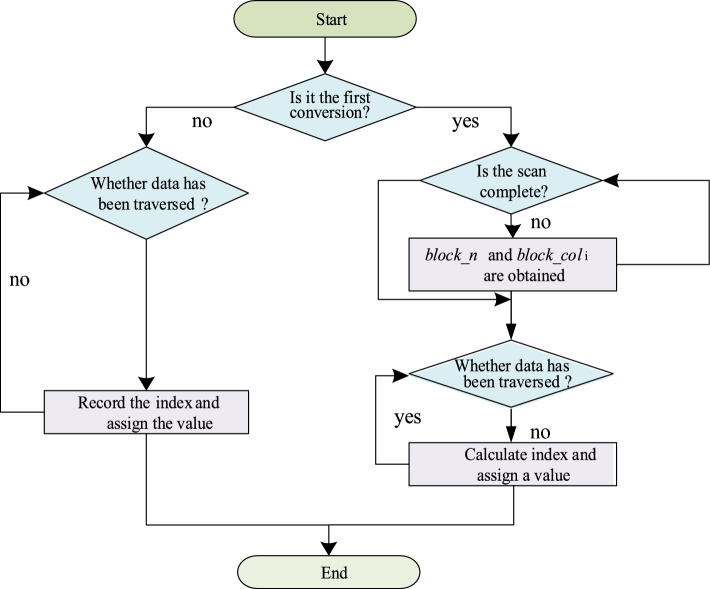
Transformation of CSR to BCSR storage format.

Finally, the map array and data array corresponded one to one, and the *val*_*index*_ of *val* where the corresponding data is stored is recorded. The specific transformation process is shown in Fig. 3.

#### Data storage transformation strategy

**Figure 4:**
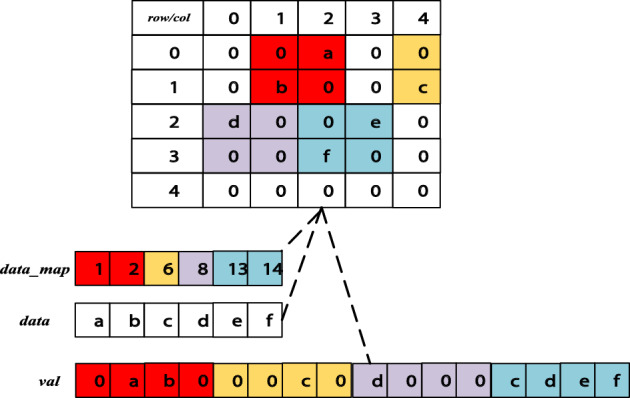
The memory data storage transformation strategy.

In practice, the sparse matrix is not computed by one operation but needs a continuous iterative process, and each iteration will update the value of the non-zero element and vector *x* in the matrix. To further improve the data transformation efficiency and reduce the format transformation time, we designed a memorized data storage transformation strategy. We added the *data_map* array to the structure of BCSR storage to record the subscript of *val* array corresponding to each non-zero data after the first CSR transformation to BCSR. As shown in Fig. 4, we first determine whether it is the first conversion, if not, we can directly load the data of CSR into the corresponding *block* of BCSR when performing iterative calculation (storing the non-zero elements directly into the *val* array according to the subscripts recorded in *data_map*), eliminating the process of constantly scanning the matrix to calculate the index of non-zero elements, reducing the format conversion time and greatly improving the conversion efficiency.

### Data storage transformation strategy

Analyzing the heterogeneous structure of SW26010P many-core processor, one core (CG) of SW26010P many-core processor includes one master core (MPE) and one slave core array, and one slave core array contains 64 slave cores (CPE), which are mainly responsible for the operation of data. To make full use of the computing resources of CPE and divide the computing tasks equally among the slave cores as much as possible, this paper adopts dynamic task division to reduce the waiting time of each CPE and realize load balancing. Firstly, combined with the special structure of SW26010P many-core processor and the storage characteristics of BCSR storage format, the sparse matrix of N*N is divided into m*n task *ls_block* and numbered as follows:

$$Task = \left\{ {ls\_block_{1} ,ls\_block_{2} ,ls\_block_{3} , \ldots ls\_block_{k} } \right\}$$

The 64 CPE independently obtained data for calculation at the same time, and the starting position of each CPE to obtain data was *core*_*index*_, which was calculated according to the slave core number (*_PEN*) and the number of row-slices in the task *block (lsn)*. The specific calculation method is shown in formula (5).

5 $$core_{index} = ptr\left[ {\_PEN*\left( {ls_{n} + 63} \right)/64} \right]$$

The global index is used in task assignment, while the local index is used in CPE calculation. According to formulas (4) and (5), the conversion equation from local index to global index is shown in formula (6).

6 $$index_{global} = core_{index} + \, val_{index}$$

MPE schedules each CPE for calculation. When the CPE computes the allocated *ls_block*_*i*_, it obtains the next task *block* to be calculated from the task pool according to the task number. Because the memory of the LDM is limited, data needs to be obtained multiple times. Therefore, ensure that data must be obtained in data blocks each time.

**Figure 5:**
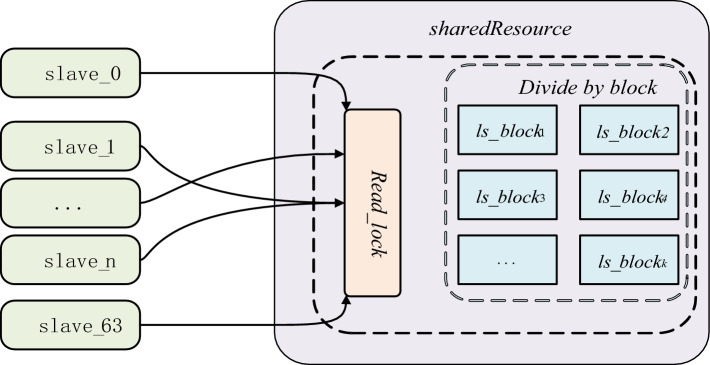
Schematic diagram of dynamic partitioning.

After the task *block* data is obtained based on the task number, the *block* number *task*_*i*_ of the calculated task *block* must be accumulated. This paper uses parallel computing, to prevent the thread from reading the wrong task, and guarantee at the same time there can be only one value of the CPE change *task*_*i*_ before the implementation accumulative operation of variable *task*_*i*_ locked, and after completion of the procedure to unlock and synchronous operation, in order to prevent the resource conflicts and repeated calculation, the specific process as shown in Fig. 5.

### Vector X access optimization strategy

**Figure 6:**
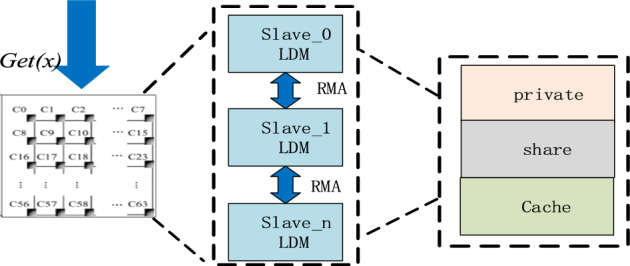
Vector x acquisition process.

It is well known that in SpMV algorithm, the vector *x* corresponding to the large-scale sparse matrix also occupies a huge space. Due to the limited LDM space of CPE, vector *x* is allocated to all CPEs. If the current coefficient vector *x* required from the kernel exists locally, it is directly obtained; If it does not exist, it will find the CPE number stored in the required vector *x*, and then obtain it from the corresponding CPE through RMA communication. The specific process is shown in Fig. 6.

In the calculation process of SpMV based on SW26010P many-core processor, the communication time occupies most of the overall time. To reduce the communication time and make the best use of LDM memory, the vector *x* access optimization strategy is improved. This algorithm is improved by referring to the idea of literature^25^. We change the static cache storage scheme, set multiple dynamic *blocks*, and adjust the size of a single dynamic *block* according to the processor’s performance. To make full use of the space and reduce communication times, we adopted the double cache strategy based on slave core architecture. We set two buffers and one static buffer *buffer*_*static*_ with a fixed size of *x*_*ssize*_, and the proportion of *x*_*ssize*_ in the LDM space is 20%. For static storage of most vector *x* for reuse; The other uses the dynamic buffer *buffer*_*dynamic*_, and the size is set to *x*_*dsize*_, which consists of multiple dynamic *blocks*. We call the first address of each CPE static space storage vector *x* as *x_ss*[*_PEN*], and the starting position of the static space vector *x* acquired by each CPE as *x*_*s*_[*_PEN*], *x*_*s*_[*_PEN*]is calculated as shown in formula (7).

7 $$x_{s} \left[ {\_PEN} \right] = \_PEN*\left( {row + 63} \right)/64$$

**Figure 7:**
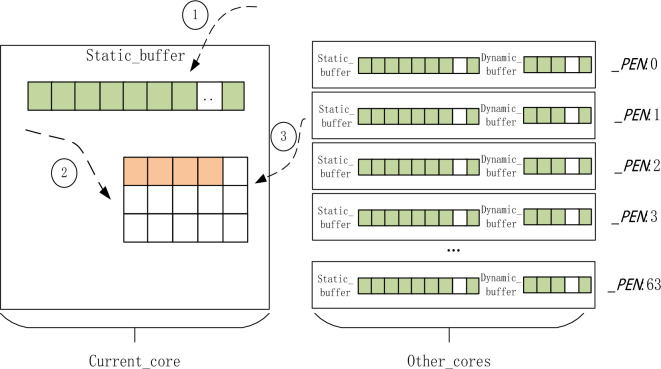
Vector x optimization algorithm.

In the calculation process, first query whether the required vector *x* is in *buffer*_*static*_, if not, then go to *buffer*_*dynamic*_ to query, if still not, then need to get the size data of a dynamic block from other slave cores to get the vector *x* by RMA, and then update *buffer*_*dynamic*_, the flow is shown in Fig. 7.

The RMA algorithm flow of CPE to realize dynamic vector *x* update among each other is as follows: Firstly, the target slave kernel number *g_id* for data acquisition is calculated as shown in formula (8), where the *index* represents the position of the required vector *x*. Then, the starting position of dynamic vector *x* is obtained as *x*_*d*_, and the calculation is shown in formula (9).

8 $$g\_id = index/\left( {row + \, 63} \right)/64$$

9 $$x_{d} = x\_ss\left[ {g\_id} \right] + index - x_{s} \left[ {g\_id} \right]$$

Once the *g_id*, *xd* is obtained, the data is stored in the *buffer*_*dynamic*_ of the current slave core through RMA and is used for calculation.

## Experiments

This section focuses on the design of experimental environment and test scheme in detail. To verify the effectiveness of the scheme, as many matrix properties as possible are covered. In this experiment, matrices of different shapes and sizes in the sparse Matrix library of Matrix Market^33^ are selected as test sets to verify the optimization of this scheme, and the experimental data are analyzed in detail.

### Experimental Environment

Matrix Market collects large sparse matrices obtained by discretizing many practical problems in science and engineering. To verify the effectiveness and universality of this scheme, a core group of the SW26010P many-core processor is used as the test platform for this experiment. The computational tasks are loaded asynchronously to the slave core for execution with the help of the high-performance threading library *Athread*. The experiment mainly analyzes and compares the two important indexes of the optimized calculation time and the master–slave speedup ratio. In addition, we consider that the *block_size* of BCSR storage format greatly impacts the calculation performance, so this scheme carries out a lot of tests and analysis on the selection of *block_size*. For the test matrix of this scheme, sparse matrices of different shapes in Matrix Market set with matrix size ranging from thousands to hundreds of thousands and the number of non-zero elements ranging from tens of thousands to millions are selected for a large number of tests.

### Experimental test and analysis

#### BCSR storage advantages

**Figure 8:**
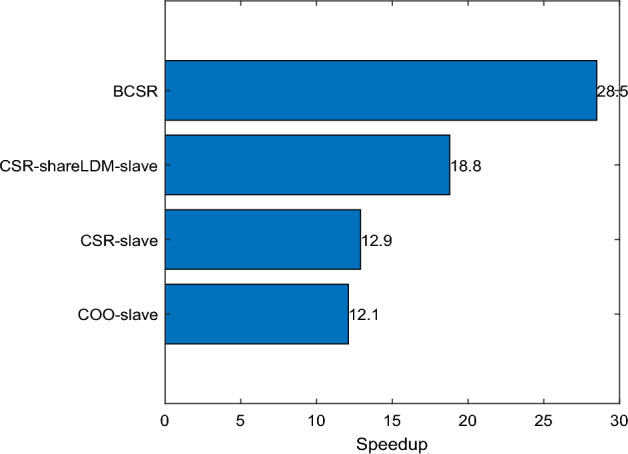
Acceleration ratios of different storage formats.

This paper employs the BCSR sparse matrix storage format, which helps to improve storage efficiency by dividing the matrix into smaller blocks, especially when the matrix has locally dense regions. Since BCSR divides the matrix into blocks of a fixed size, it can reduce the number of times vector x is fetched during the computation process, thereby enhancing memory computation efficiency. To demonstrate the effectiveness of the scheme, tests were conducted using the classic matrix in the pimplefoam solver from the OpenFoam software, with a matrix size of 9216*9216 and 61,696 non-zero elements. Experiments were conducted using CSR format, COO format, BCSR format, and so on. In particular, an optimized CSR format data storage was designed, called the CSR-shareLDM-slave scheme, which used a shared LDM approach to store matrix data for testing, with results as shown in Fig. 8. The experimental results prove that the proposed BCSR format can achieve the best acceleration ratio, with an acceleration ratio of up to 28.5, effectively improving the speed of fluid dynamics simulations.

**Figure 9:**
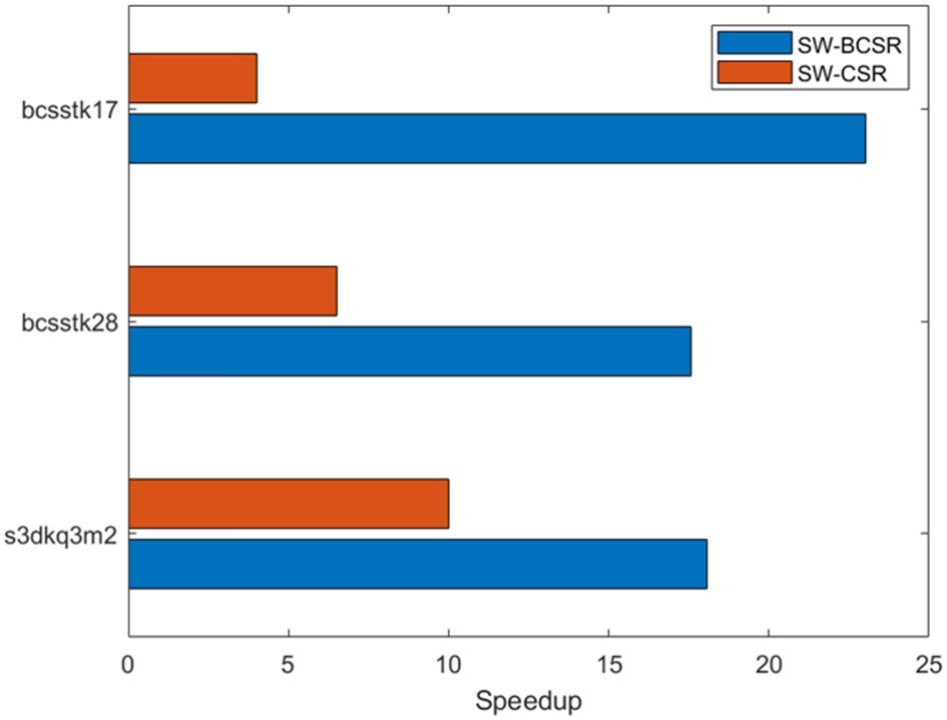
BCSR’s acceleration effect on SW26010P processor.

This paper implements the SpMV computation under the BCSR storage format on the domestically produced Sunway supercomputer, with a very noticeable acceleration effect. Currently, there is still relatively little work based on the new generation of domestic Sunway supercomputers. To prove the effectiveness of this method, a comparison was made with the CSR format SpMV on the Sunway TaihuLight supercomputer as presented in literature^28^. Three large-scale sparse matrices were selected for comparative experiments, and the results are shown in Fig. 9. The results show that among the three matrices, the BCSR storage format proposed in this paper has a significant advantage, with the maximum acceleration ratio reaching 23.03, which is a very good optimization effect.

#### Master–slave speedup ratio test

**Figure 10:**
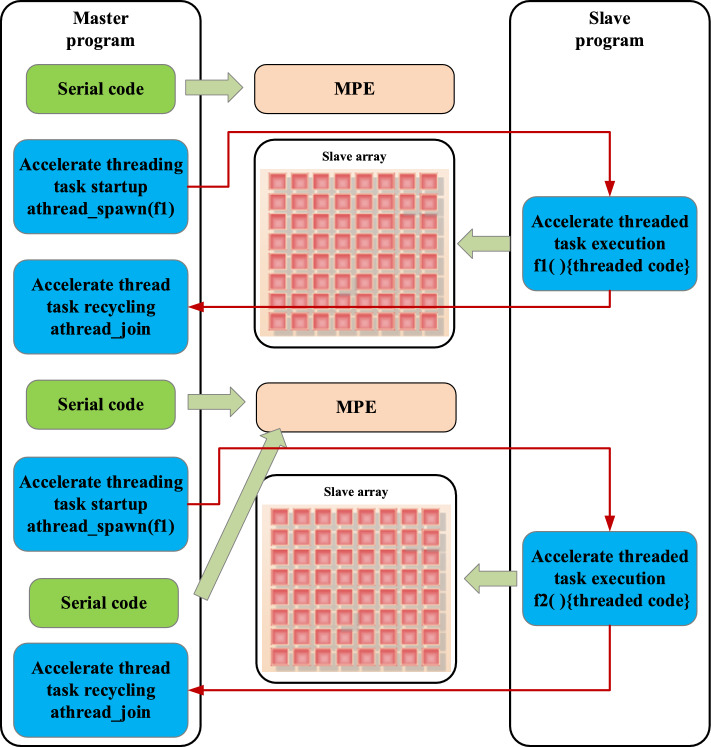
Sunway supercomputing’s heterogeneous acceleration programming model.

The article uses the self-developed SW26010P many-core processor from China as the experimental platform. This processor is composed of 6 core groups (CGs), each containing 1 computational control core (MPE) and 64 computational cores (CPE). The processor adopts a heterogeneous acceleration programming model, as shown in Fig. 10. The MPE is responsible for the distribution and management of data and accelerated tasks, using the relevant interfaces of athread to manage the acceleration thread tasks. Depending on the different functionalities of the interfaces, the accelerated thread tasks will be executed on one or more thread arrays. The CPE mainly realizes the acceleration of the core functional modules.

If 6 CGs are used for computation, since the inter-group process-level parallelism is carried out through MPI, the matrix data and vectors need to be evenly divided according to 6 processes, which may result in the data required for computation within process A being present within process B. Inter-process data transfer takes a long time and occupies a lot of memory, leading to low CPU utilization. For matrices at the scale of tens of millions, using one core group (CG) for computation can greatly reduce the time required for data transmission, thus optimizing performance.

Speedup is the ratio of the time consumed by the same computation task in many-core processors and single-core processors. It measures the performance and effect of parallelization of parallel programs. In the field of parallel computing, the speedup ratio is one of the important indexes to measure the performance of parallel processing. The computational performance of SpMV is affected by many factors, such as the shape of the sparse matrix, the degree of data sparsity, and the size of the matrix. To test the effectiveness and universality of the proposed scheme, sparse matrices are firstly simply classified according to size, shape and size. Then the calculation time and speedup ratio are tested. Detailed parameters of the sparse matrix selected in this scheme are shown in Table 1.

**Table 1:** The first set of test matrices.

NO	Name	Size	Non-zero element
1	add20	2395 * 2395	17,319
2	getmat12	4929 * 4929	33,044
3	orsirr_1	1030 * 1030	6858
4	af23560	23,560 * 23,560	460,598
5	bcsstk21	3600 * 3600	15,100
6	e30r0000	9661 * 9661	306,356
7	bcsstk18	11,948 * 11,948	149,090
8	bcsstk28	4410 * 4410	111,717
9	bcsstk18	11,948 * 11,948	219,024
10	bcsstk17	10,974 * 10,974	219,812

**Figure 11:**
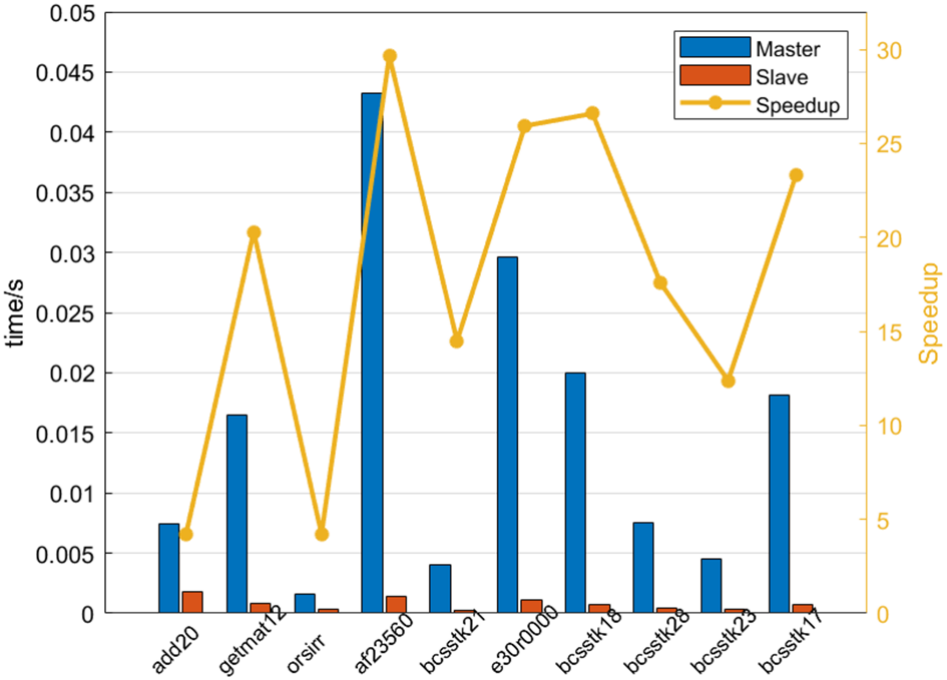
The results of the first set of test matrix.

We use the control variable method, set the *block_size* of all the test matrices to 5*5, and then count the computation time of all the sparse matrices. To avoid errors, we calculated the average value for 20 times and obtained the acceleration ratio according to the calculated results. The statistical results are shown in Fig. 11.

Based on the results of the first set of tests, it is easy to conclude as follows: In the selected 10 sparse matrix samples, the computational performance of our scheme in this paper is significantly improved compared with the traditional computational methods. However, due to the difference in sparse matrix shape, matrix scale and element sparsity, the master–slave speedup ratio is also different. In this group of examples, the master–slave acceleration ratio is 28.81 times, and the average value is 18.83 times, which has a good acceleration effect and universality.

**Figure 12:**
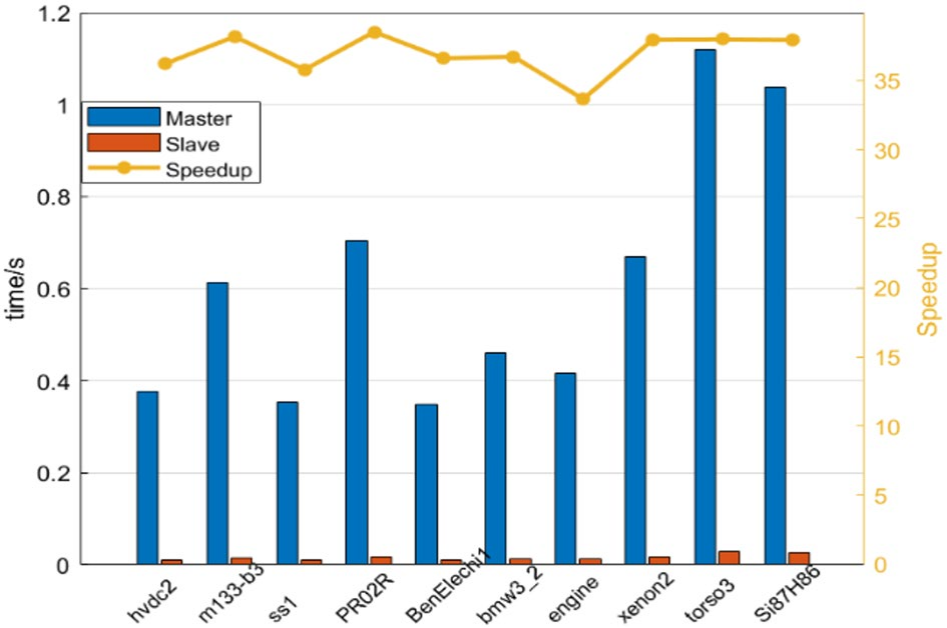
The results of the second set of test matrix.

To further test the effectiveness of this scheme, we reselected 10 larger sparse matrices from the Matrix Market as the second group of test samples, and the detailed parameters of the selected sparse matrices are shown in Table 2. The matrix size of the selected second group of test samples is larger and the number of non-zero elements can reach millions. Similarly, we used the control variable method, set the *block_size* of all test matrices to 5*5, and then counted the computation time of sparse matrices in the test set. To avoid errors, we also adopted the strategy of calculating the average value for 20 times and finally calculated the speedup ratio according to the calculated results. The statistical results are shown in Fig. 12.

**Table 2:** The second set of test matrices.

NO	Name	Size	Non-zero element
1	hvdc2	189,960 * 189,860	1,347,273
2	m133-b3	200,200 * 200,200	800,800
3	ss1	205,280 * 205,282	845,089
4	PR02R	161,070 * 161,070	8,185,136
5	BenElechi1	245,874 * 245,874	6,698,185
6	bmw3_2	227,362 * 227,362	5,757,996
7	engine	143,571 * 143,571	2,424,822
8	xenon2	259,156 * 259,156	3,866,688
9	torso3	259,156 * 259,156	4,429,042
10	Si87H86	240,369 * 240,369	5,451,000

Based on the results of the second set of tests, it is easy to conclude as follows: In the selected 10 sparse matrix samples, the computational performance of our scheme in this paper is significantly improved compared with the traditional computational methods, and the acceleration effect is more noticeable compared with the matrix selected by the first group. In the test samples of this group, the master–slave acceleration ratio of this scheme is up to 38.51 times, and the average value is up to 36.95 times, which has an excellent acceleration effect. It can be seen that the acceleration effect of this scheme is more evident for large matrix sizes and many non-zero elements.

#### The selection of block_size

In order to achieve the best performance of sparse matrix–vector multiplication, the *block_size* plays an essential role in BCSR storage format. The distribution of non-zero elements of sparse matrix affects the best selection of *block_size*. To test the impact of *block_size* on the optimization performance, we randomly selected a group of sparse matrices from the Matrix Market and tested them under three different *block_size*. The specific parameters of the sparse matrix and *block_size* are shown in Table 3.

**Table 3:** Sparse matrix block_size selection table.

No	Name	block_size1	block_size2	block_size3
1	hvdc2	5 * 5	8 * 8	12 * 12
2	m133-b3	5 * 5	8 * 8	12 * 12
3	ss1	5 * 5	8 * 8	12 * 12
4	PR02R	5 * 5	8 * 8	12 * 12
5	BenElechi1	5 * 5	8 * 8	12 * 12
6	bmw3_2	5 * 5	8 * 8	12 * 12
7	engine	5 * 5	8 * 8	12 * 12
8	xenon2	5 * 5	8 * 8	12 * 12
9	torso3	5 * 5	8 * 8	12 * 12
10	Si87H86	5 * 5	8 * 8	12 * 12

**Figure 13:**
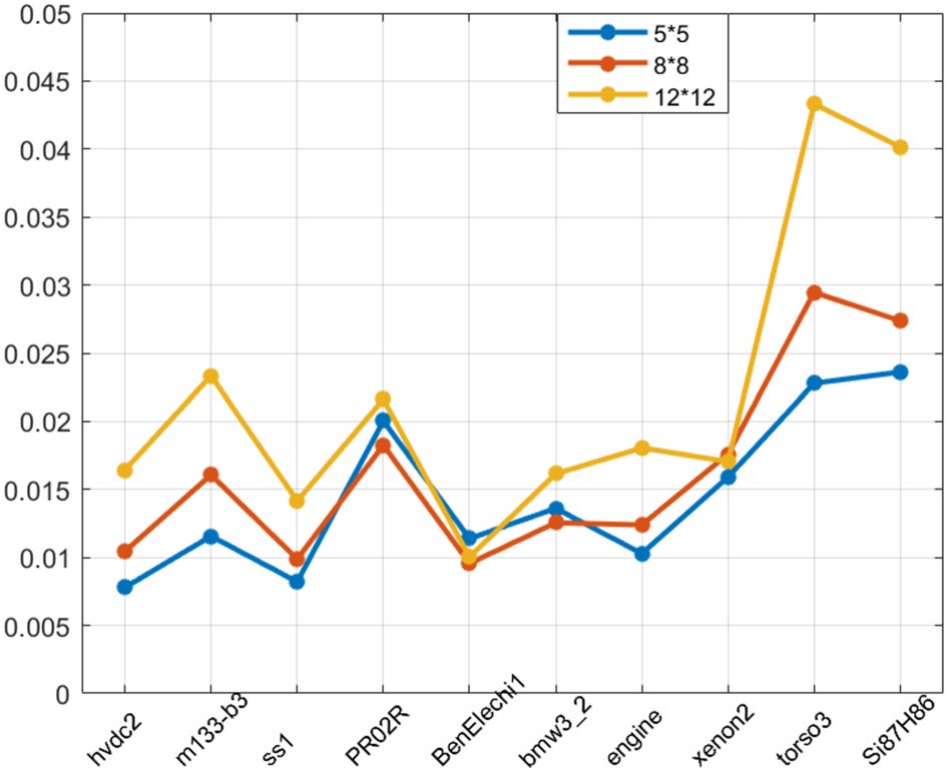
The block_size selection test.

Through the sparse matrix selected in this experiment, we successively select *block_size*1, *block_size*2 and *block_size*3 for execution and then calculate the computation time of the sparse matrix under different *block_sizes*. In addition, in order to avoid errors, we also take the method of calculating the average value for 20 times. The statistical results are shown in Fig. 13.

From the test results, it can be concluded that *block_size* has a great impact on the calculation performance of SpMV. As shown in Fig. 13, when *block_size* is 5*5, the calculation performance is the best. This is because the density of the selected sparse matrix is low. If the *block_size* is too large, a large number of invalid calculations will be carried out to reduce the calculation performance. Therefore, selecting the appropriate *block_size* for SpMV optimization based on BCSR storage format is very important. The sampling rate is the proportion of the selected matrix samples to the total size of the matrix. Through experiments, we conclude that when the sampling rate reaches a certain value, the *block_size* obtained in most cases will be stable, and the selection result of *block_size* will not be changed even if the sampling rate continues to be increased. Therefore, the estimation method can be used in practical applications to obtain the optimal *block_size* value.

## Conclusions

In this paper, we propose a SpMV algorithm based on BCSR storage format for SW26010P many-core processor. In order to quickly transform the matrix in CSR storage format into BCSR storage, we design a memorized data storage transformation strategy, which dramatically reduces the transformation time and improves the efficiency of data transformation. In addition, by studying the master–slave architecture of SW26010P many-core processor, we adopted the dynamic task scheduling method to realize the load balance between each CPE during computation and reduce the waiting time between each CPE. To improve the utilization of LDM storage space and the hit ratio of vector *x*, we divide the LDM memory space reasonably, and improve the LDM dual cache optimization strategy to further improve the SpMV computing performance. To verify the effectiveness of our work, we selected a large number of representative sparse matrices of different shapes and sizes from the Matrix Market for testing. The results show that the method in this paper has an obvious acceleration effect on sparse matrices of various sizes and sizes, and the effect is more evident for large matrices, the master–slave acceleration ratio can be up to 38 times, which has a significant optimization effect. The optimization method used in this paper has implications for other complex applications of SW26010P many-core processor. More significantly, this is of great significance to the software ecological construction and performance optimization of self-developed supercomputers. Finally, there are still many shortcomings in our work, such as how to adaptively select the size of *block_size* and how to improve the hit rate of vector *x* further.

## Data Availability

The data that support the results of this study are available from the Matrix Market, and can be accessed via the website https://math.nist.gov/MatrixMarket/.The majority of the data comes from “https://math.nist.gov/MatrixMarket/extreme.html” and “https://math.nist.gov/MatrixMarket/searchtool.html” of this website.
